# How are medical devices regulated in the European Union?

**DOI:** 10.1258/jrsm.2012.120036

**Published:** 2012-04

**Authors:** Elaine French-Mowat, Joanne Burnett

**Affiliations:** 1 Guildford Medical Device Evaluation Centre (GMEC), The Royal Surrey County Hospital, Egerton Road, Guildford, Surrey GU2 7XX; 2 NICE - MTEP, Level 1A City Tower Piccadilly Plaza, Manchester M1 4BD, UK

## Background

The Medical Technology Evaluation Programme (MTEP) within the National Institute for Health and Clinical Excellence (NICE) was set up in 2009; this is a programme focusing specifically on the selection and evaluation of new or innovative medical technologies (including devices and diagnostics).

One of the requirements to enable a product to be evaluated by the MTEP is that the device is CE marked or it will be CE marked within twelve months. This report was produced to describe the CE marking process for different categories of medical technology and the types/quality/quantity of evidence that are required for each category.

## Summary

Medical devices cannot be placed on the European market without conforming to the strict safety requirements of the European Union; one of these requirements is the affixation of the CE conformity mark. This article is an overview of the CE marking process only; it is not a document that should be referred to on its own. All manufacturers wishing to gain a CE mark should refer to the official documents.

## Legislation

Collectively known as the Medical Device Directive (MDD), this core legal framework consists of three directives that regulate the safety and marketing of medical devices in Europe and came into effect in the 1990s. The three directives are the:

- Active Implantable Medical Device Directive (AIMDD 90/385/EE);^1^
- Medical Device Directive (MDD 93/42/EEC);^2^
- In Vitro Diagnostic Medical Device Directive (IVDMDD 98/79/EC).^3^

These have been supplemented since by several necessary updates, due to new and emerging technologies which have challenged the current framework, highlighted gaps and pointed to a certain scarcity of expertise.^4^ The MDD (article 1.2a) define a Medical Device as:

‘Any instrument, apparatus, appliance, material or other article, whether used alone or in combination, including software necessary for its proper application intended by the manufacturer to be used for human beings for the purpose of:

- Diagnosis, prevention, monitoring, treatment or alleviation of disease;
- Diagnosis, monitoring, treatment, alleviation of or compensation for an injury or disability;
- Investigation, replacement or modification of the anatomy or of a physiological process;
- Control of conception.

and which does not achieve its principal intended action in or on the human body by pharmacological, immunological or metabolic means, but which may be assisted in its function by such means.’

## The CE mark

This is a conformity mark which all European medical devices must have before they can be marketed. It is seen as a declaration by the manufacturer that the product meets all the appropriate provisions of the relevant legislation including those related to safety and, where required, has been assessed in accordance with these. Article 1, MDD 93/42/EEC, states:

1. ‘Devices, other than devices which are custom-made or intended for clinical investigations, considered to meet the essential requirements referred to in Article 3, must bear the CE marking of conformity when they are placed on the market.
2. The CE marking of conformity, as shown in Annex XII, must appear in a visible, legible and indelible form on the device or its sterile pack, where practicable and appropriate, and on the instructions for use. Where applicable, the CE marking must also appear on the sales packaging.
  It shall be accompanied by the identification number of the notified body responsible for implementation of the procedures set out in Annexes II, IV, V and VI.
3. It is prohibited to affix marks or inscriptions which are likely to mislead third parties with regard to the meaning or the graphics of the CE marking. Any other mark may be affixed to the device, to the packaging or to the instruction leaflet accompanying the device, provided that the visibility and legibility of the CE marking is not thereby reduced.’

The CE conformity mark consists of the initials ‘CE’ taking the following form:

The various components of the CE marking must have substantially the same vertical dimension, which may not be less than 5 mm, although this minimum dimension may be waived for small-scale devices.

The initials “CE” do not stand for any specific words; it is a symbol that is seen as a declaration by the manufacturer that the product meets all the appropriate provisions of the relevant legislation. This includes those related to safety and it shows that, where required, the device has been assessed in accordance with the appropriate procedures. The CE mark also means that the product can be freely marketed anywhere in the European Economic Area (EEA) without further control.

## Overview of CE marking process

Competent Authorities (CA), Notified Bodies (NB) and authorized representatives are all involved in the CE marking process. CA's exist in each European Member State and are nominated by each government to monitor and ensure compliance with its provisions of the MDD. The CA designates a NB to ensure that conformity assessment procedures are completed according to the relevant criteria. The authorized representative, designated by the manufacturers, is legally responsible for compliance with the regulations and acts as the first point of contact for the EU authorities.

It is up to the manufacturer to ensure that their product complies with the essential requirements of the relevant EU legislation. A general overview of the CE marking process is:

- Check which directives and Annexes apply;
- Choose conformity assessment procedure/route;
- Prepare design dossier (for required devices);
- Prepare technical documentation;
- Prepare declaration of conformity;
- Submit to notified body (NB), if applicable, for certification;
- Register with CA (by manufacturer or an authorised representative);
- Apply CE marking and market product;
- Implement vigilance and post market surveillance by monitoring safety and efficiency, and reviewing experience of use and any action required.

Irrespective of the class of the device, all devices must:

- Meet the essential requirements, including the requirements regarding the information to be supplied by the manufacturer;
- Evaluate clinical efficacy and any side effects, if applicable by means of a pre-clinical and clinical evaluation;
- Be subject to the reporting requirements under the medical device vigilance system;
- Be CE marked (except accepted exemptions);
- Be registered with the CA where the manufacturer (or the authorised representative) has a registered place of business.

### Device classification

All medical devices are placed into one of four graduated categories, using the classification rules listed in Directive 93/42/EEC Annex IX.^2^ It is considered more feasible, economically and justifiably, to categorize medical devices rather than all of them being subject to the rigorous conformity assessment procedures.

The categories are Class I (including Is & Im), Class IIa and IIb and Class III, with Class III ranked as the highest. The higher the classification the greater the level of assessment required by NBs. It is the intended purpose of the device that determines the classification and not the particular technical characteristics. Considerations for classification include the duration of contact with the body, degree of invasiveness and local versus systemic effect. The highest possible class applies if a device can be classified according to several rules. Table 1 has details for each class.^2^

**Table 1:** Examples of product classification

	Classification	Risk	Description	Examples
	General controls			Hospital beds, bed pans
I	Sterile (Is)	Low	Most non-invasive devices that do not interact with the body.	Sterile plasters
	Measuring (Im)			Thermometers, weighing scales.
IIa	Special controls required: may include special labelling, mandatory performance standards & post-market surveillance	Medium	Exchange energy with a patient in a therapeutic manner or are used to diagnose or monitor medical conditions. Generally invasive but limited to natural orifices, if hazardous to a patient then it becomes a class IIb	Powered wheelchairs, hearing aids, ultrasonic diagnostic equipment
IIb	Special controls (as IIa)	Medium	Most surgically invasive/active devices partially or totally implantable in the body. May modify composition of body fluids.	Infusion pumps, ventilators, surgical lasers
III	Pre-market approval is the required process of scientific review to ensure the safety and effectiveness of these	High	Support or sustain human life and are of substantial importance in preventing impairment of human health, or which present a potential, unreasonable risk of illness or injury. Device that connects directly with the Central Circulatory System or CNS, or contains a medicinal product.	Many implants: vascular & neurological, replacement heart valves, silicone gel-filled breast implants, and implanted cerebella stimulators

### Conformity assessment procedure

**Figure 1:**
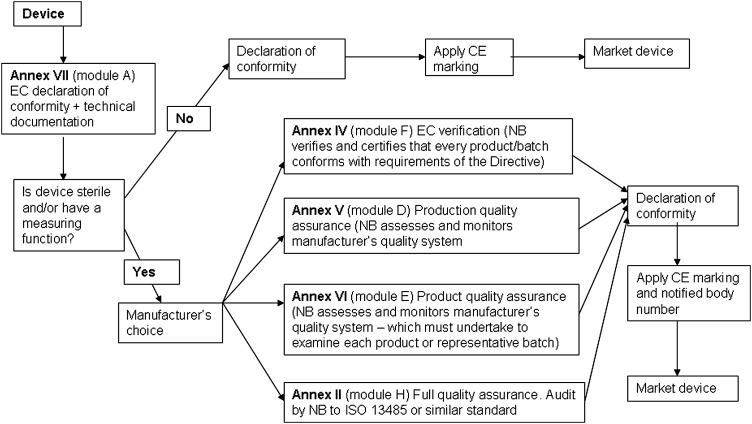
Class I conformity assessment procedures

**Figure 2:**
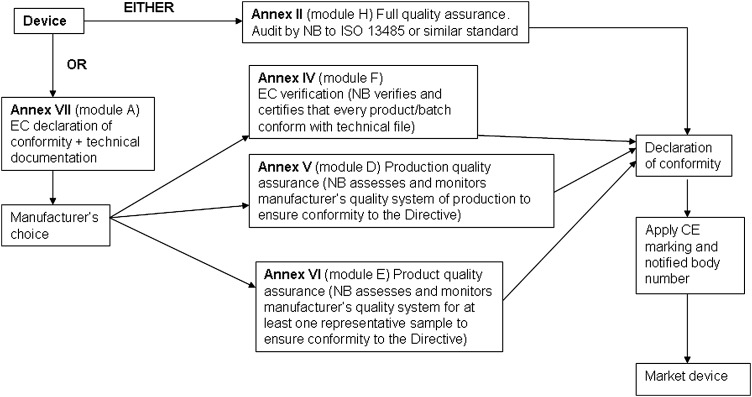
Class IIa conformity assessment procedures

**Figure 3:**
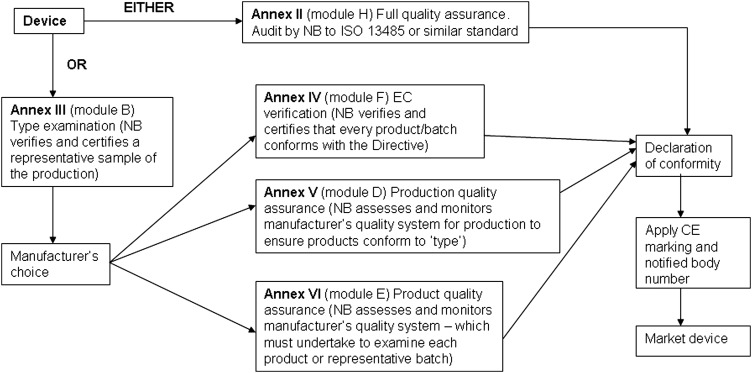
Class IIb conformity assessment procedures

**Figure 4:**
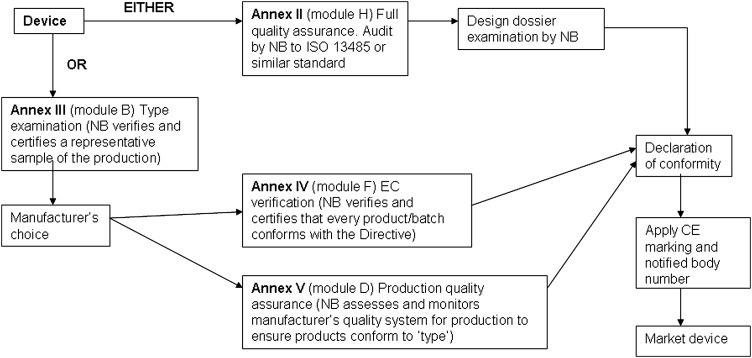
Class III conformity assessment procedures

A conformity assessment procedure demonstrates that the device complies with the requirements of Directive 93/42/EEC.^2^ Compliance is stated by establishing a Conformity Declaration. The classification of the device dictates the appropriate conformity assessment procedure. In some cases the manufacturer has a choice of conformity route. The conformity assessment routes are shown in Figures 1, 2, 3 and 4.

#### Class I

This route is self-declaration or self-certification and is described in Annex VII Module A, EC Declaration of Conformity. The manufacturer ensures and formally declares, via a written statement, that the products meet the applicable provisions of the Directive.

#### Class IIa

The manufacturer declares conformity with the provisions of the Directive and Regulations (Annex VII) and ensures that the products comply with relevant essential requirements. However, for Class IIa products, this declaration must be backed up in all cases with conformity assessment by a NB using Annex II, IV, V or VI.

#### Class IIb

Manufacturers of Class IIb devices may also choose the full quality assurance route (Annex II) including assessment by a NB of the technical documentation for at least one representative sample for each generic device group for compliance with the Directive (Annex II section 7).

#### Class III

This route to conformity is similar to those for Class IIb devices but additionally requires the manufacturer to submit the design dossier to the NB for approval under audit of the full quality assurance system (Annex II) and do not allow the Annex VI option.

Active implantable medical devices (AIMD) and *in vitro* diagnostic medical devices (IVD) are also subject to conformity procedures.

### Required documentation

For five years (15 years for implantable devices) after the final production of the device, the declaration of conformity, the technical documentation, the design dossier (if applicable) and the NB decisions and reports/certificates must be kept at the disposal of the CA.

As part of the compliance strategy, manufacturers also have to provide evidence of basic good manufacturing practice (GMP).^5,6^ The GMP or Formal Quality System applies to devices in Class Is, Im, IIa, IIb and III, AIMDs and IVDs listed in Annex II and those for self-testing. Basic Class I medical devices (non-measuring and not supplied sterile) and general IVDs (IVDs that are not for self-testing and/or do not appear in Annex II) do not need a formal or full quality system, basic GMP is sufficient.

#### Declaration of conformity

This document is required for all medical devices, AIMDs and IVDs, and is a written statement produced by the manufacturer to formally declare that a named product conforms to the appropriate directives. If more than one Directive is applicable then these should all be listed on the declaration. It also identifies who is responsible for a product and provides a first point of contact if there are any problems to be investigated. It must be kept in the technical file.

The declaration of conformity should have the name and address of manufacturer, identification of the product allowing traceability, list of relevant directives, declaration statement, name and position/job title of person signing. This should be someone with enough responsibility to ensure the declaration is true which is affirmed by their signature and date.

#### Technical documentation and design dossier

These have to be produced to allow the assessment of the conformity with the essential requirements of the Directive; the level and complexity of detail will depend on the conformity assessment route. Overall it should cover the design, manufacture and intended operation of the product. The ‘operation’ of the product includes installation, preparation for use, pre-use checks and maintenance, calibration and servicing as appropriate to the particular medical device. Data has to be provided that is sufficient to demonstrate that the device will perform safely and achieve the stated performance claims for its intended use. The technical documentation must include:

- A general description of the product, including any variants planned and its intended use(s);
- Design drawings, methods of manufacture envisaged and diagrams of components, sub-assemblies, circuits;
- The descriptions and explanations necessary to understand the above mentioned drawings and diagrams and the operations of the product;
- Results of the risk analysis and a list of the harmonized standards, applied in full or in part, and descriptions of the solutions adopted to meet the essential requirements;
- In the case of products placed on the market in a sterile condition, description of the methods used and the validation report;
- The results of the design calculations and of the inspections carried out;
- The solutions adopted to ensure that the design and construction conform to safety principles;
- The pre-clinical evaluation;
- The clinical evaluation in accordance with Annex X;
- The label and instructions for use.

The technical documentation and quality management system (QMS) are audited annually by the NB. The NB must also periodically carry out appropriate inspections and assessments to ensure the manufacturer applies the approved QMS and produce a report. They may also carry out unannounced visits to check that the QMS is working properly and produce a report on their findings. The manufacturer must inform the NB of any changes applied to the QMS.

#### Notified bodies evidence

Any reports or decisions issued by the NB should be kept in the technical file. These may include surveillance, inspection, test and clinical evaluation assessment reports or an approval of changes decision as outlined below.

For basic class I devices, only the declaration of conformity is required before affixing the CE marking and placing the device on the market. All other devices are required to be certified by third parties. These third parties are laboratories; inspection and the certification bodies are the NBs. Following a successful conformity assessment, the NB issues an EC certificate indicating the route to certification. The manufacturer, having signed a declaration of conformity, can then legally place the CE marked product on the European market.

### Quality of evidence required

Technical files and design dossiers are viewed as controlled documents and as such should be part of the manufacturer's quality system with systems in place to update.

The manufacturer should be able to demonstrate where and how the documentation is held and maintained. The pages should be numbered and the information be presented in an organized, concise and coherent manner to facilitate review by the NB, if applicable, as conclusions and synopsis. Tables and flow charts are effective mechanisms to provide summary results. Generally, documents that demonstrate compliance with the essential requirements are summarized in the text of the technical file or design dossier and included as an attachment or appendix.

## Medical technology not requiring a CE mark

Although the rules will adequately classify the vast majority of existing devices, a small number of products may be more difficult to classify. If a manufacturer is unsure how its devices should be classified, it should first consult a NB. If doubts remain or there is a disagreement with the NB, the relevant CA should be approached in accordance with Article 9 of Directive 93/42/EEC.^2^ Exemptions from CE marking include:

- Custom-made devices;
- Devices intended for clinical investigation;
- Health protection – urgent unusual circumstances, humanitarian use;
- In-house use.

## Other relevant legislation

Each medical device can only be placed on the market and/or put into service when the product complies with the provisions of all applicable directives and when the conformity assessment has been carried out in accordance with all the directives. As a result, several directives may have to be taken into consideration for one product; these include: General Product Safety Directive (92/59/EEC),^7^ Medical Electrical Equipment (BS EN 60601) and the Directive of Electromagnetic Compatibility (2004/108/EC).^8^

## DECLARATIONS

### Competing interests

The authors have no competing interests to declare. All articles were originally commissioned by NICE from independent External Assessment Centres (EACs) to address areas of interest to the Medical Technologies Evaluation Programme (MTEP, originally known as Evaluation Pathways EP). The EACs were engaged under a contract to NICE

### Funding

NICE

### Ethical approval

Not applicable

### Guarantor

Chris Pomfrett

### Contributorship

Elaine French-Mowatt wrote the first version of the manuscript while working for GMEC, an independent academic External Assessment Centre working under contract to NICE.

Joanne Burnett extensively edited and updated the manuscript in order for it to be suitable for publication. Joanne Burnett is a Technical Analyst working in the Medical Technologies Evaluation Programme (MTEP) of NICE.

Dr Chris Pomfrett does not claim authorship, but acted as supervising editor for this work in NICE. Dr Chris Pomfrett is a Technical Adviser working in MTEP at NICE

### Acknowledgments

None
